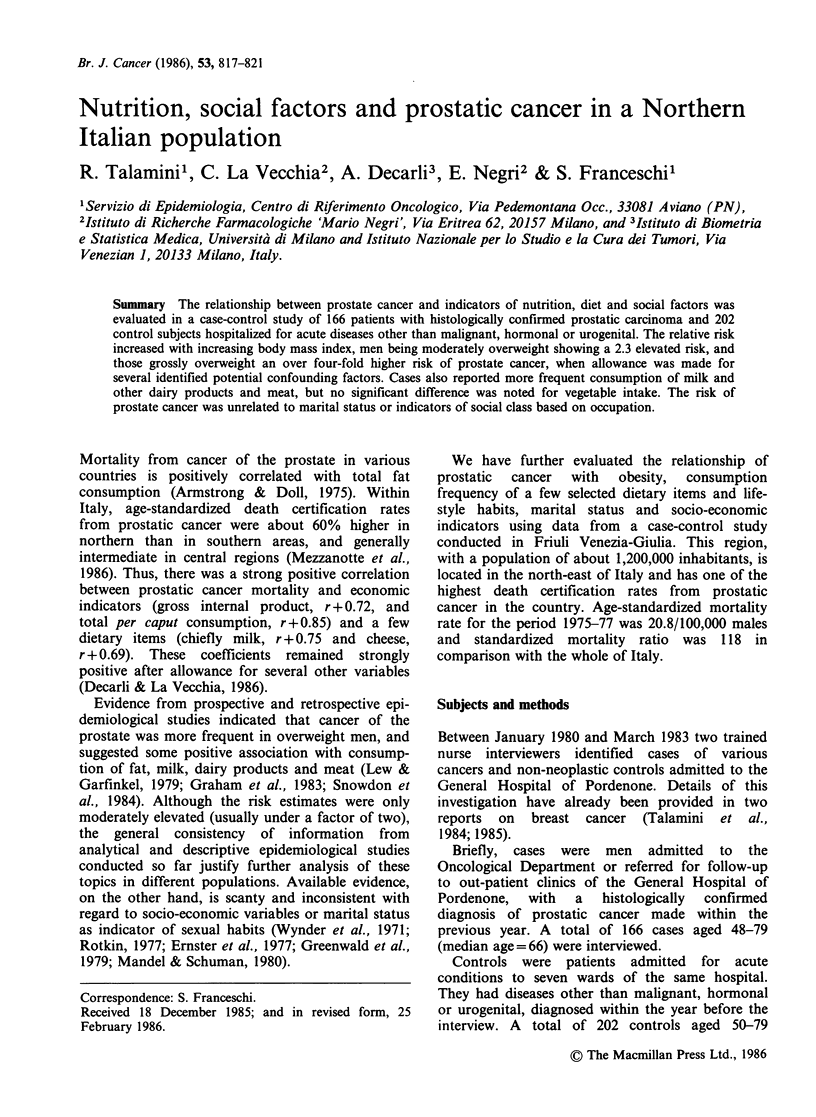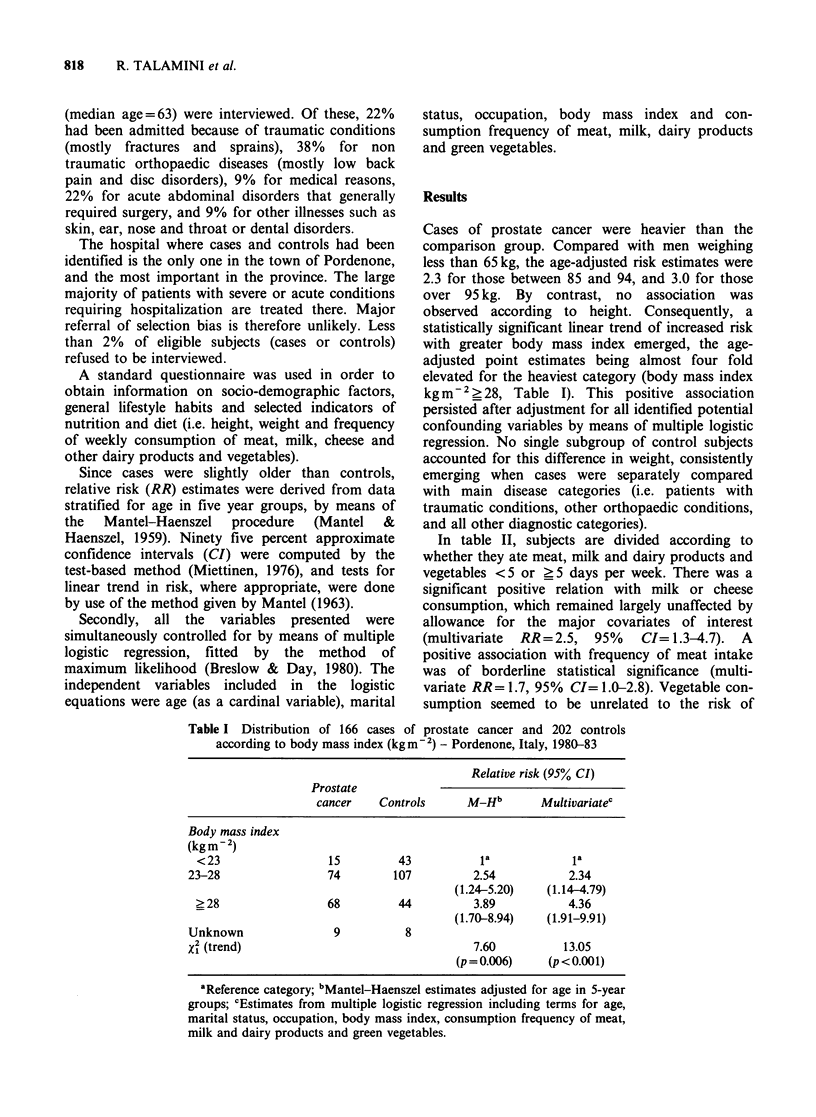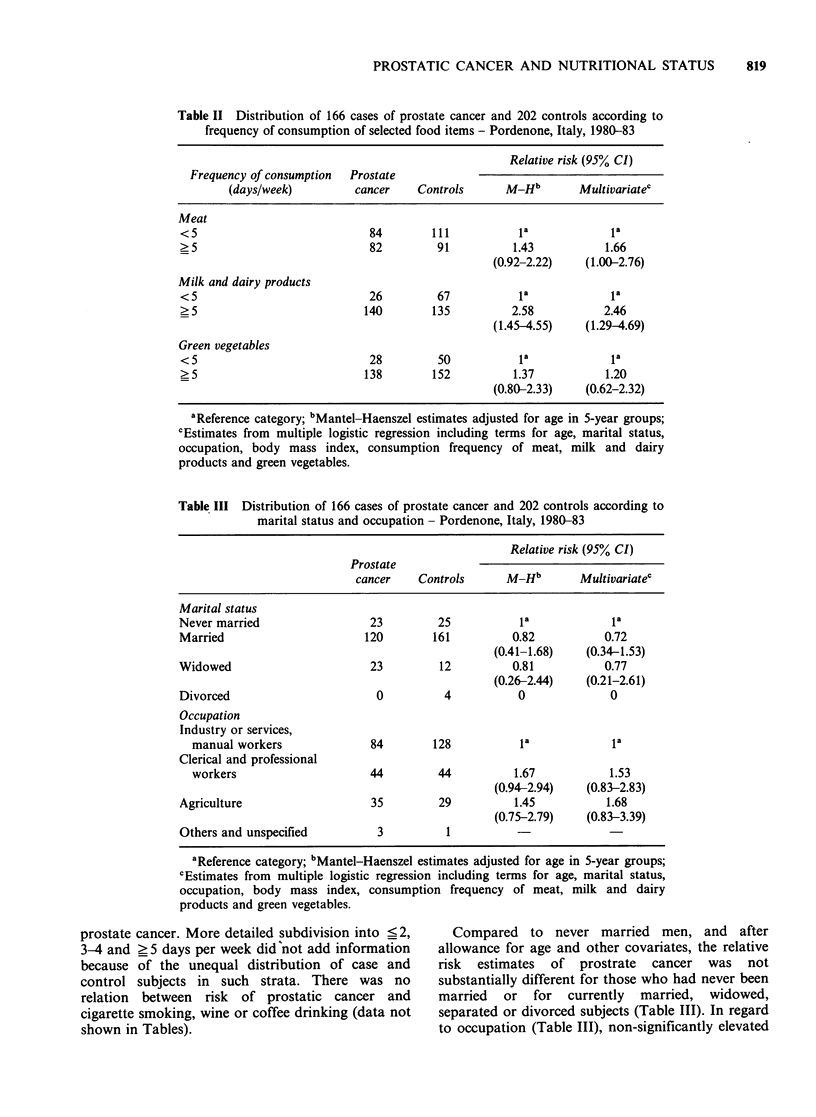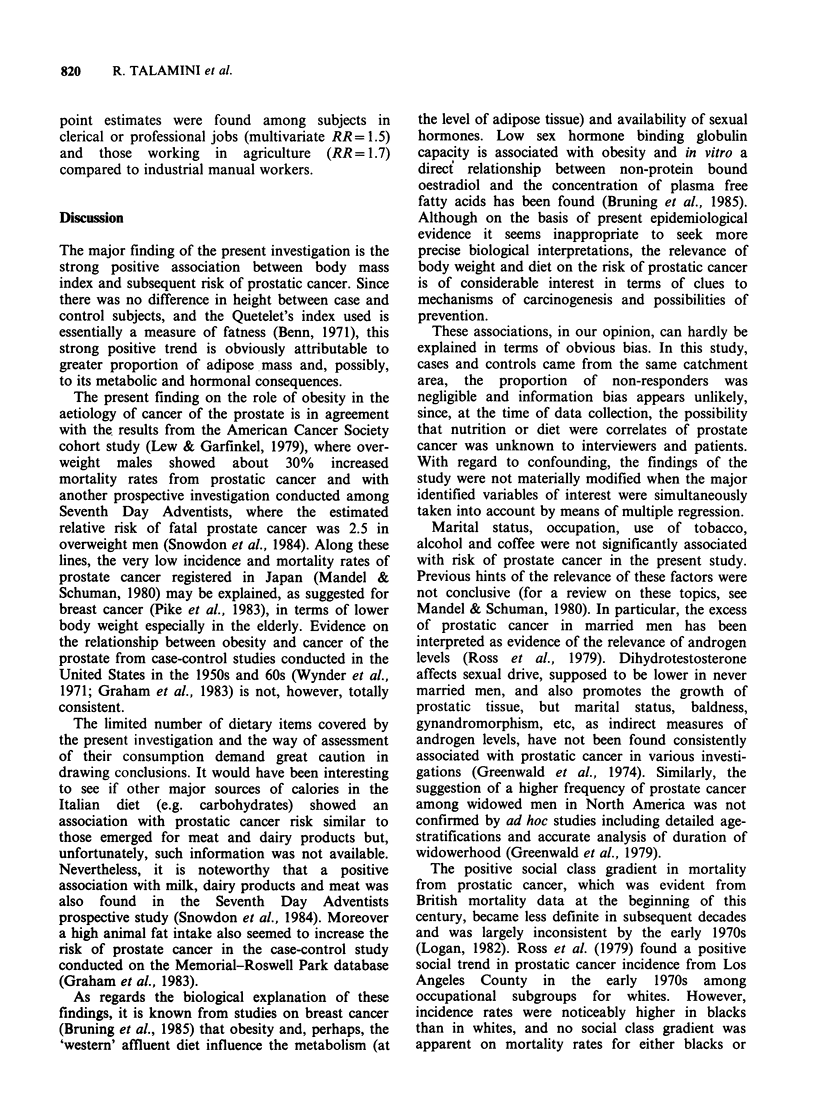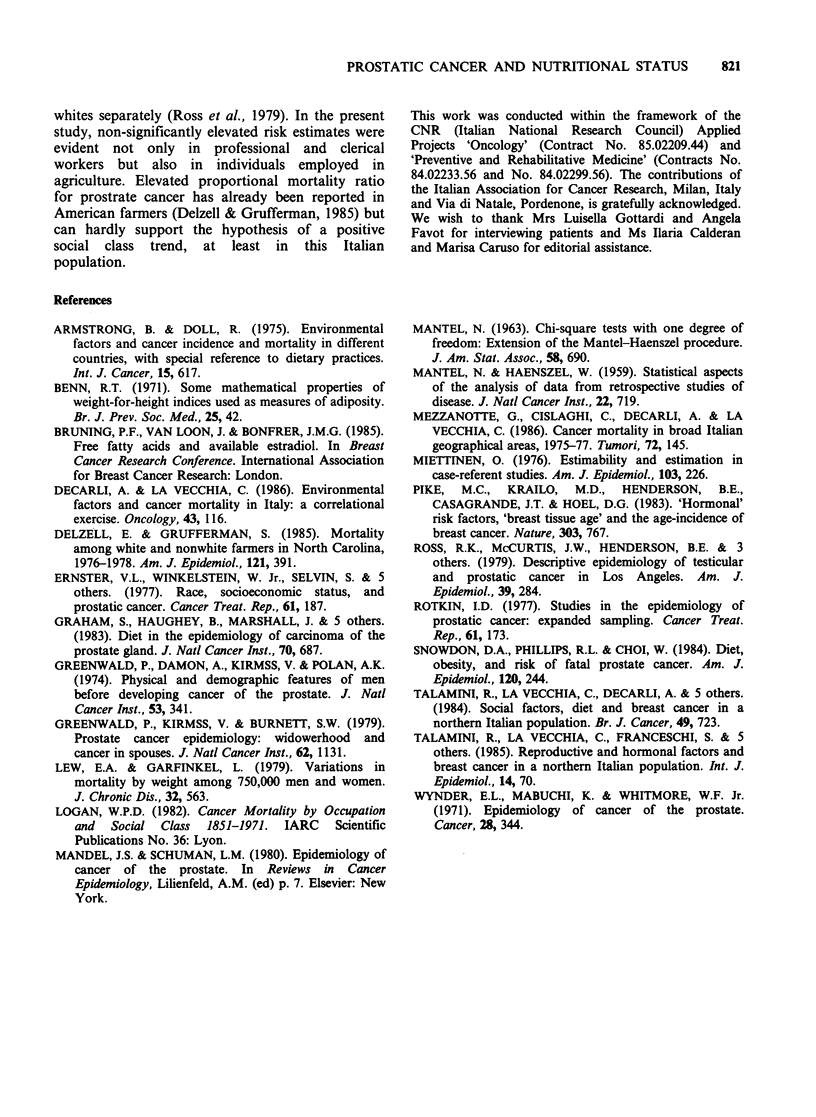# Nutrition, social factors and prostatic cancer in a Northern Italian population.

**DOI:** 10.1038/bjc.1986.138

**Published:** 1986-06

**Authors:** R. Talamini, C. La Vecchia, A. Decarli, E. Negri, S. Franceschi

## Abstract

The relationship between prostate cancer and indicators of nutrition, diet and social factors was evaluated in a case-control study of 166 patients with histologically confirmed prostatic carcinoma and 202 control subjects hospitalized for acute diseases other than malignant, hormonal or urogenital. The relative risk increased with increasing body mass index, men being moderately overweight showing a 2.3 elevated risk, and those grossly overweight an over four-fold higher risk of prostate cancer, when allowance was made for several identified potential confounding factors. Cases also reported more frequent consumption of milk and other dairy products and meat, but no significant difference was noted for vegetable intake. The risk of prostate cancer was unrelated to marital status or indicators of social class based on occupation.


Br. J. Cancer (1986), 53, 817–821

### Nutrition, social factors and prostatic cancer in a Northern Italian population

R. Talamini[1], C. La Vecchia[2], A. Decarli[3], E. Negri[2] & S. Franceschi[1]

[1]*Servizio di Epidemiologia, Centro di Riferimento Oncologico, Via Pedemontana Occ., 33081 Aviano (PN),* [2]*Istituto di Richerche Farmacologiche 'Mario Negri', Via Eritrea 62, 20157 Milano, and* [3]*Istituto di Biometria e Statistica Medica, Università di Milano and Istituto Nazionale per lo Studio e la Cura dei Tumori, Via Venezian 1, 20133 Milano, Italy.*

**Summary** The relationship between prostate cancer and indicators of nutrition, diet and social factors was evaluated in a case-control study of 166 patients with histologically confirmed prostatic carcinoma and 202 control subjects hospitalized for acute diseases other than malignant, hormonal or urogenital. The relative risk increased with increasing body mass index, men being moderately overweight showing a 2.3 elevated risk, and those grossly overweight an over four-fold higher risk of prostate cancer, when allowance was made for several identified potential confounding factors. Cases also reported more frequent consumption of milk and other dairy products and meat, but no significant difference was noted for vegetable intake. The risk of prostate cancer was unrelated to marital status or indicators of social class based on occupation.

Mortality from cancer of the prostate in various countries is positively correlated with total fat consumption (Armstrong & Doll, 1975). Within Italy, age-standardized death certification rates from prostatic cancer were about 60% higher in northern than in southern areas, and generally intermediate in central regions (Mezzanotte *et al.*, 1986). Thus, there was a strong positive correlation between prostatic cancer mortality and economic indicators (gross internal product, $r+0.72$, and total *per caput* consumption, $r+0.85$) and a few dietary items (chiefly milk, $r+0.75$ and cheese, $r+0.69$). These coefficients remained strongly positive after allowance for several other variables (Decarli & La Vecchia, 1986).

Evidence from prospective and retrospective epidemiological studies indicated that cancer of the prostate was more frequent in overweight men, and suggested some positive association with consumption of fat, milk, dairy products and meat (Lew & Garfinkel, 1979; Graham *et al.*, 1983; Snowdon *et al.*, 1984). Although the risk estimates were only moderately elevated (usually under a factor of two), the general consistency of information from analytical and descriptive epidemiological studies conducted so far justify further analysis of these topics in different populations. Available evidence, on the other hand, is scanty and inconsistent with regard to socio-economic variables or marital status as indicator of sexual habits (Wynder *et al.*, 1971; Rotkin, 1977; Ernster *et al.*, 1977; Greenwald *et al.*, 1979; Mandel & Schuman, 1980).

Correspondence: S. Franceschi.
Received 18 December 1985; and in revised form, 25 February 1986.

We have further evaluated the relationship of prostatic cancer with obesity, consumption frequency of a few selected dietary items and life-style habits, marital status and socio-economic indicators using data from a case-control study conducted in Friuli Venezia-Giulia. This region, with a population of about 1,200,000 inhabitants, is located in the north-east of Italy and has one of the highest death certification rates from prostatic cancer in the country. Age-standardized mortality rate for the period 1975–77 was 20.8/100,000 males and standardized mortality ratio was 118 in comparison with the whole of Italy.

### Subjects and methods

Between January 1980 and March 1983 two trained nurse interviewers identified cases of various cancers and non-neoplastic controls admitted to the General Hospital of Pordenone. Details of this investigation have already been provided in two reports on breast cancer (Talamini *et al.*, 1984; 1985).

Briefly, cases were men admitted to the Oncological Department or referred for follow-up to out-patient clinics of the General Hospital of Pordenone, with a histologically confirmed diagnosis of prostatic cancer made within the previous year. A total of 166 cases aged 48–79 (median age = 66) were interviewed.

Controls were patients admitted for acute conditions to seven wards of the same hospital. They had diseases other than malignant, hormonal or urogenital, diagnosed within the year before the interview. A total of 202 controls aged 50–79

© The Macmillan Press Ltd., 1986

(median age = 63) were interviewed. Of these, 22% had been admitted because of traumatic conditions (mostly fractures and sprains), 38% for non traumatic orthopaedic diseases (mostly low back pain and disc disorders), 9% for medical reasons, 22% for acute abdominal disorders that generally required surgery, and 9% for other illnesses such as skin, ear, nose and throat or dental disorders.

The hospital where cases and controls had been identified is the only one in the town of Pordenone, and the most important in the province. The large majority of patients with severe or acute conditions requiring hospitalization are treated there. Major referral of selection bias is therefore unlikely. Less than 2% of eligible subjects (cases or controls) refused to be interviewed.

A standard questionnaire was used in order to obtain information on socio-demographic factors, general lifestyle habits and selected indicators of nutrition and diet (i.e. height, weight and frequency of weekly consumption of meat, milk, cheese and other dairy products and vegetables).

Since cases were slightly older than controls, relative risk (*RR*) estimates were derived from data stratified for age in five year groups, by means of the Mantel–Haenszel procedure (Mantel & Haenszel, 1959). Ninety five percent approximate confidence intervals (*CI*) were computed by the test-based method (Miettinen, 1976), and tests for linear trend in risk, where appropriate, were done by use of the method given by Mantel (1963).

Secondly, all the variables presented were simultaneously controlled for by means of multiple logistic regression, fitted by the method of maximum likelihood (Breslow & Day, 1980). The independent variables included in the logistic equations were age (as a cardinal variable), marital status, occupation, body mass index and consumption frequency of meat, milk, dairy products and green vegetables.

### Results

Cases of prostate cancer were heavier than the comparison group. Compared with men weighing less than 65 kg, the age-adjusted risk estimates were 2.3 for those between 85 and 94, and 3.0 for those over 95 kg. By contrast, no association was observed according to height. Consequently, a statistically significant linear trend of increased risk with greater body mass index emerged, the age-adjusted point estimates being almost four fold elevated for the heaviest category (body mass index $kg\,m^{-2} \geq 28$, Table I). This positive association persisted after adjustment for all identified potential confounding variables by means of multiple logistic regression. No single subgroup of control subjects accounted for this difference in weight, consistently emerging when cases were separately compared with main disease categories (i.e. patients with traumatic conditions, other orthopaedic conditions, and all other diagnostic categories).

In table II, subjects are divided according to whether they ate meat, milk and dairy products and vegetables <5 or ≥5 days per week. There was a significant positive relation with milk or cheese consumption, which remained largely unaffected by allowance for the major covariates of interest (multivariate *RR* = 2.5, 95% *CI* = 1.3–4.7). A positive association with frequency of meat intake was of borderline statistical significance (multivariate *RR* = 1.7, 95% *CI* = 1.0–2.8). Vegetable consumption seemed to be unrelated to the risk of

**Table I** Distribution of 166 cases of prostate cancer and 202 controls according to body mass index ($kg\,m^{-2}$) – Pordenone, Italy, 1980–83

| | Prostate cancer | Controls | Relative risk (95% CI) | |
|---|---|---|---|---|
| | | | *M–H*[b] | *Multivariate*[c] |
| *Body mass index* ($kg\,m^{-2}$) | | | | |
| <23 | 15 | 43 | 1[a] | 1[a] |
| 23–28 | 74 | 107 | 2.54 (1.24–5.20) | 2.34 (1.14–4.79) |
| ≥28 | 68 | 44 | 3.89 (1.70–8.94) | 4.36 (1.91–9.91) |
| Unknown | 9 | 8 | | |
| $\chi_1^2$ (trend) | | | 7.60 ($p = 0.006$) | 13.05 ($p < 0.001$) |

[a]Reference category; [b]Mantel–Haenszel estimates adjusted for age in 5-year groups; [c]Estimates from multiple logistic regression including terms for age, marital status, occupation, body mass index, consumption frequency of meat, milk and dairy products and green vegetables.

**Table II** Distribution of 166 cases of prostate cancer and 202 controls according to frequency of consumption of selected food items – Pordenone, Italy, 1980–83

| *Frequency of consumption (days/week)* | *Prostate cancer* | *Controls* | *Relative risk (95% CI)* | |
|---|---|---|---|---|
| | | | *M–H*[b] | *Multivariate*[c] |
| *Meat* | | | | |
| <5 | 84 | 111 | 1[a] | 1[a] |
| ≧5 | 82 | 91 | 1.43 (0.92–2.22) | 1.66 (1.00–2.76) |
| *Milk and dairy products* | | | | |
| <5 | 26 | 67 | 1[a] | 1[a] |
| ≧5 | 140 | 135 | 2.58 (1.45–4.55) | 2.46 (1.29–4.69) |
| *Green vegetables* | | | | |
| <5 | 28 | 50 | 1[a] | 1[a] |
| ≧5 | 138 | 152 | 1.37 (0.80–2.33) | 1.20 (0.62–2.32) |

[a]Reference category; [b]Mantel–Haenszel estimates adjusted for age in 5-year groups; [c]Estimates from multiple logistic regression including terms for age, marital status, occupation, body mass index, consumption frequency of meat, milk and dairy products and green vegetables.

**Table III** Distribution of 166 cases of prostate cancer and 202 controls according to marital status and occupation – Pordenone, Italy, 1980–83

| | *Prostate cancer* | *Controls* | *Relative risk (95% CI)* | |
|---|---|---|---|---|
| | | | *M–H*[b] | *Multivariate*[c] |
| *Marital status* | | | | |
| Never married | 23 | 25 | 1[a] | 1[a] |
| Married | 120 | 161 | 0.82 (0.41–1.68) | 0.72 (0.34–1.53) |
| Widowed | 23 | 12 | 0.81 (0.26–2.44) | 0.77 (0.21–2.61) |
| Divorced | 0 | 4 | 0 | 0 |
| *Occupation* | | | | |
| Industry or services, manual workers | 84 | 128 | 1[a] | 1[a] |
| Clerical and professional workers | 44 | 44 | 1.67 (0.94–2.94) | 1.53 (0.83–2.83) |
| Agriculture | 35 | 29 | 1.45 (0.75–2.79) | 1.68 (0.83–3.39) |
| Others and unspecified | 3 | 1 | — | — |

[a]Reference category; [b]Mantel–Haenszel estimates adjusted for age in 5-year groups; [c]Estimates from multiple logistic regression including terms for age, marital status, occupation, body mass index, consumption frequency of meat, milk and dairy products and green vegetables.

prostate cancer. More detailed subdivision into ≦2, 3–4 and ≧5 days per week did not add information because of the unequal distribution of case and control subjects in such strata. There was no relation between risk of prostatic cancer and cigarette smoking, wine or coffee drinking (data not shown in Tables).

Compared to never married men, and after allowance for age and other covariates, the relative risk estimates of prostrate cancer was not substantially different for those who had never been married or for currently married, widowed, separated or divorced subjects (Table III). In regard to occupation (Table III), non-significantly elevated point estimates were found among subjects in clerical or professional jobs (multivariate $RR = 1.5$) and those working in agriculture ($RR = 1.7$) compared to industrial manual workers.

### Discussion

The major finding of the present investigation is the strong positive association between body mass index and subsequent risk of prostatic cancer. Since there was no difference in height between case and control subjects, and the Quetelet's index used is essentially a measure of fatness (Benn, 1971), this strong positive trend is obviously attributable to greater proportion of adipose mass and, possibly, to its metabolic and hormonal consequences.

The present finding on the role of obesity in the aetiology of cancer of the prostate is in agreement with the results from the American Cancer Society cohort study (Lew & Garfinkel, 1979), where overweight males showed about 30% increased mortality rates from prostatic cancer and with another prospective investigation conducted among Seventh Day Adventists, where the estimated relative risk of fatal prostate cancer was 2.5 in overweight men (Snowdon *et al.*, 1984). Along these lines, the very low incidence and mortality rates of prostate cancer registered in Japan (Mandel & Schuman, 1980) may be explained, as suggested for breast cancer (Pike *et al.*, 1983), in terms of lower body weight especially in the elderly. Evidence on the relationship between obesity and cancer of the prostate from case-control studies conducted in the United States in the 1950s and 60s (Wynder *et al.*, 1971; Graham *et al.*, 1983) is not, however, totally consistent.

The limited number of dietary items covered by the present investigation and the way of assessment of their consumption demand great caution in drawing conclusions. It would have been interesting to see if other major sources of calories in the Italian diet (e.g. carbohydrates) showed an association with prostatic cancer risk similar to those emerged for meat and dairy products but, unfortunately, such information was not available. Nevertheless, it is noteworthy that a positive association with milk, dairy products and meat was also found in the Seventh Day Adventists prospective study (Snowdon *et al.*, 1984). Moreover a high animal fat intake also seemed to increase the risk of prostate cancer in the case-control study conducted on the Memorial–Roswell Park database (Graham *et al.*, 1983).

As regards the biological explanation of these findings, it is known from studies on breast cancer (Bruning *et al.*, 1985) that obesity and, perhaps, the 'western' affluent diet influence the metabolism (at the level of adipose tissue) and availability of sexual hormones. Low sex hormone binding globulin capacity is associated with obesity and *in vitro* a direct relationship between non-protein bound oestradiol and the concentration of plasma free fatty acids has been found (Bruning *et al.*, 1985). Although on the basis of present epidemiological evidence it seems inappropriate to seek more precise biological interpretations, the relevance of body weight and diet on the risk of prostatic cancer is of considerable interest in terms of clues to mechanisms of carcinogenesis and possibilities of prevention.

These associations, in our opinion, can hardly be explained in terms of obvious bias. In this study, cases and controls came from the same catchment area, the proportion of non-responders was negligible and information bias appears unlikely, since, at the time of data collection, the possibility that nutrition or diet were correlates of prostate cancer was unknown to interviewers and patients. With regard to confounding, the findings of the study were not materially modified when the major identified variables of interest were simultaneously taken into account by means of multiple regression.

Marital status, occupation, use of tobacco, alcohol and coffee were not significantly associated with risk of prostate cancer in the present study. Previous hints of the relevance of these factors were not conclusive (for a review on these topics, see Mandel & Schuman, 1980). In particular, the excess of prostatic cancer in married men has been interpreted as evidence of the relevance of androgen levels (Ross *et al.*, 1979). Dihydrotestosterone affects sexual drive, supposed to be lower in never married men, and also promotes the growth of prostatic tissue, but marital status, baldness, gynandromorphism, etc, as indirect measures of androgen levels, have not been found consistently associated with prostatic cancer in various investigations (Greenwald *et al.*, 1974). Similarly, the suggestion of a higher frequency of prostate cancer among widowed men in North America was not confirmed by *ad hoc* studies including detailed age-stratifications and accurate analysis of duration of widowerhood (Greenwald *et al.*, 1979).

The positive social class gradient in mortality from prostatic cancer, which was evident from British mortality data at the beginning of this century, became less definite in subsequent decades and was largely inconsistent by the early 1970s (Logan, 1982). Ross *et al.* (1979) found a positive social trend in prostatic cancer incidence from Los Angeles County in the early 1970s among occupational subgroups for whites. However, incidence rates were noticeably higher in blacks than in whites, and no social class gradient was apparent on mortality rates for either blacks or whites separately (Ross *et al.*, 1979). In the present study, non-significantly elevated risk estimates were evident not only in professional and clerical workers but also in individuals employed in agriculture. Elevated proportional mortality ratio for prostrate cancer has already been reported in American farmers (Delzell & Grufferman, 1985) but can hardly support the hypothesis of a positive social class trend, at least in this Italian population.

This work was conducted within the framework of the CNR (Italian National Research Council) Applied Projects 'Oncology' (Contract No. 85.02209.44) and 'Preventive and Rehabilitative Medicine' (Contracts No. 84.02233.56 and No. 84.02299.56). The contributions of the Italian Association for Cancer Research, Milan, Italy and Via di Natale, Pordenone, is gratefully acknowledged. We wish to thank Mrs Luisella Gottardi and Angela Favot for interviewing patients and Ms Ilaria Calderan and Marisa Caruso for editorial assistance.

### References

ARMSTRONG, B. & DOLL, R. (1975). Environmental factors and cancer incidence and mortality in different countries, with special reference to dietary practices. *Int. J. Cancer*, **15**, 617.

BENN, R.T. (1971). Some mathematical properties of weight-for-height indices used as measures of adiposity. *Br. J. Prev. Soc. Med.*, **25**, 42.

BRUNING, P.F., VAN LOON, J. & BONFRER, J.M.G. (1985). Free fatty acids and available estradiol. In *Breast Cancer Research Conference*. International Association for Breast Cancer Research: London.

DECARLI, A. & LA VECCHIA, C. (1986). Environmental factors and cancer mortality in Italy: a correlational exercise. *Oncology*, **43**, 116.

DELZELL, E. & GRUFFERMAN, S. (1985). Mortality among white and nonwhite farmers in North Carolina, 1976–1978. *Am. J. Epidemiol.*, **121**, 391.

ERNSTER, V.L., WINKELSTEIN, W. Jr., SELVIN, S. & 5 others. (1977). Race, socioeconomic status, and prostatic cancer. *Cancer Treat. Rep.*, **61**, 187.

GRAHAM, S., HAUGHEY, B., MARSHALL, J. & 5 others. (1983). Diet in the epidemiology of carcinoma of the prostate gland. *J. Natl Cancer Inst.*, **70**, 687.

GREENWALD, P., DAMON, A., KIRMSS, V. & POLAN, A.K. (1974). Physical and demographic features of men before developing cancer of the prostate. *J. Natl Cancer Inst.*, **53**, 341.

GREENWALD, P., KIRMSS, V. & BURNETT, S.W. (1979). Prostate cancer epidemiology: widowerhood and cancer in spouses. *J. Natl Cancer Inst.*, **62**, 1131.

LEW, E.A. & GARFINKEL, L. (1979). Variations in mortality by weight among 750,000 men and women. *J. Chronic Dis.*, **32**, 563.

LOGAN, W.P.D. (1982). *Cancer Mortality by Occupation and Social Class 1851–1971*. IARC Scientific Publications No. 36: Lyon.

MANDEL, J.S. & SCHUMAN, L.M. (1980). Epidemiology of cancer of the prostate. In *Reviews in Cancer Epidemiology*, Lilienfeld, A.M. (ed) p. 7. Elsevier: New York.

MANTEL, N. (1963). Chi-square tests with one degree of freedom: Extension of the Mantel–Haenszel procedure. *J. Am. Stat. Assoc.*, **58**, 690.

MANTEL, N. & HAENSZEL, W. (1959). Statistical aspects of the analysis of data from retrospective studies of disease. *J. Natl Cancer Inst.*, **22**, 719.

MEZZANOTTE, G., CISLAGHI, C., DECARLI, A. & LA VECCHIA, C. (1986). Cancer mortality in broad Italian geographical areas, 1975–77. *Tumori*, **72**, 145.

MIETTINEN, O. (1976). Estimability and estimation in case-referent studies. *Am. J. Epidemiol.*, **103**, 226.

PIKE, M.C., KRAILO, M.D., HENDERSON, B.E., CASAGRANDE, J.T. & HOEL, D.G. (1983). 'Hormonal' risk factors, 'breast tissue age' and the age-incidence of breast cancer. *Nature*, **303**, 767.

ROSS, R.K., McCURTIS, J.W., HENDERSON, B.E. & 3 others. (1979). Descriptive epidemiology of testicular and prostatic cancer in Los Angeles. *Am. J. Epidemiol.*, **39**, 284.

ROTKIN, I.D. (1977). Studies in the epidemiology of prostatic cancer: expanded sampling. *Cancer Treat. Rep.*, **61**, 173.

SNOWDON, D.A., PHILLIPS, R.L. & CHOI, W. (1984). Diet, obesity, and risk of fatal prostate cancer. *Am. J. Epidemiol.*, **120**, 244.

TALAMINI, R., LA VECCHIA, C., DECARLI, A. & 5 others. (1984). Social factors, diet and breast cancer in a northern Italian population. *Br. J. Cancer*, **49**, 723.

TALAMINI, R., LA VECCHIA, C., FRANCESCHI, S. & 5 others. (1985). Reproductive and hormonal factors and breast cancer in a northern Italian population. *Int. J. Epidemiol.*, **14**, 70.

WYNDER, E.L., MABUCHI, K. & WHITMORE, W.F. Jr. (1971). Epidemiology of cancer of the prostate. *Cancer*, **28**, 344.